# Lack of adjunctive effect of 0.1% sodium hypochlorite mouthwash combined to full‐mouth ultrasonic debridement on supragingival plaque, gingival inflammation, and subgingival microbiota: A randomized placebo‐controlled 6‐month trial

**DOI:** 10.1002/cre2.60

**Published:** 2017-03-31

**Authors:** Laís Christina Pontes Espíndola, Ana Paula Vieira Colombo

**Affiliations:** ^1^ Department of Clinics, School of Dentistry Federal University of Rio de Janeiro Brazil; ^2^ Department of Medical Microbiology, Institute of Microbiology Federal University of Rio de Janeiro Brazil

**Keywords:** dental plaque, gingivitis, mouthwash, sodium hypochlorite

## Abstract

To test the adjunctive effect of 0.1% sodium hypochlorite (NaOCl) mouthwash combined to full‐mouth ultrasonic debridement (FMUD) on reducing supragingival plaque, gingival inflammation, and microbial pathogens. In this 6‐month double‐blinded randomized clinical trial, individuals with gingivitis were assigned to test (*n* = 16) or placebo group (*n* = 16) and received FMUD followed by rinsing with 0.1% NaOCl (test) or distilled water (placebo), respectively, twice a day for 1 month. Full‐mouth periodontal examination was performed at baseline, 1, 3, and 6 months posttherapy, and subgingival plaque samples were obtained at the same time points and analysed for their composition by checkerboard. Differences between groups over time were examined by Student *t* test, Mann–Whitney, generalized linear model, and Friedman and chi‐square tests. Both therapeutic protocols resulted in significant clinical improvement in periodontal parameters over time, except for probing depth and attachment level, which had a slight mean increase of 0.2 mm (*p* < .01). No significant differences between groups were observed for any clinical parameter (*p* > .05). Most species (>65%) decreased similarly in levels in both groups over time. Significant reductions in the microbial complexes were seen mainly at 1 and 3 months, but they returned to baseline levels in both groups, except for the red and yellow complexes, and other oral species, which were kept in low levels at 6 months (*p* < .05). A 0.1% NaOCl mouthwash did not provide additional benefits to FMUD in reducing supragingival plaque, gingivitis, and/or microbial pathogens.

## 1 INTRODUCTION

Periodontal diseases are inflammatory conditions of infectious etiology that affect millions of people worldwide (Dye, 2012; Kassebaum et al., 2014), being the second major cause of tooth loss (Petersen & Ogawa, 2005). The onset and progression of these diseases involve complex interactions between commensal and pathogenic species of the periodontal microbiota and the host (Socransky & Haffajee, 2002; Socransky & Haffajee, 2005; Socransky et al., 1998). In addition, these interactions are modulated by immunological, genetic, and environmental factors that influence the onset, progression, and severity of these infections (Page et al., 1997; Silva et al., 2015). Treatment of periodontal diseases focuses mainly on suppressing the periodontal pathogenic microbiota, modulating the host response, and promoting tissue healing to provide a healthy periodontal microenvironment for the restoration of a host‐compatible microbiota (Haffajee, Teles, & Socransky, 2006; Teles, Haffajee, & Socransky, 2006). The key factor for a successful periodontal therapy is the establishment of an adequate mechanical control of dental plaque (Haffajee et al., 2003; Loe, Theilade, & Jensen, 1965; Ximenez‐Fyvie et al., 2000). However, an efficient mechanical plaque control is not achieved by most of the individuals of the general population (Ohrn & Sanz, 2009; Slots, 2012), and the introduction of antimicrobials as adjuncts to mechanical therapy has been indicated in order to improve oral hygiene (Addy, 2008; Baehni & Takeuchi, 2003; Hujoel et al., 2005; Teles & Teles, 2008; Wu & Savitt, 2002). Considering the spread of antibiotic resistance and the high costs of new generation antibiotics, antiseptics have been utilized as major antimicrobials for chemical plaque control. Sodium hypochlorite (NaOCl) is one example of a potent, safe, and inexpensive antiseptic used for decades in dentistry, particularly as an efficient irrigating solution in endodontic treatment (Mohammadi, 2008; Zehnder, 2006). NaOCl has excellent antimicrobial properties, good penetration in the polysaccharide plaque matrix, and low occurrence of side effects (Bruch, 2007; Rutala et al., 1998), and it is generally available at very low cost. However, very few studies have investigated the adjunctive effect of NaOCl as a supplement for mechanical plaque control in supportive periodontal therapy (Bizzarro, Van der Velden, & Loos, 2016; De Nardo et al., 2012; Galvan et al., 2014; Gonzalez et al., 2015; Lobene et al., 1972). The Council of the American Dental Association (ADA) on Dental Therapeutics appointed NaOCl at 0.1% as a safe and efficient antimicrobial mouthwash and suggested its use for direct application to mucous membranes (ADA, 1984). Evidence have shown that NaOCl exerts an effective antimicrobial activity against dental plaque and reduces gingival inflammation (Chávez de Paz, Bergenholtz, & Svensäter, 2010; De Nardo et al., 2012; Galvan et al., 2014; Gonzalez et al., 2015; Gosau, Hahnel, & Schwarz, 2010). Given the limited number of controlled clinical studies, the high antimicrobial efficacy and safety, as well as the low cost of NaOCl, the present investigation assessed the adjunctive effects of a 0.1% NaOCl mouthwash combined to full‐mouth ultrasonic debridement (FMUD) on reducing supragingival plaque, gingival inflammation, and levels of microbial pathogens in the subgingival microbiota of individuals with gingivitis for a 6‐month follow‐up period.

## 2 MATERIAL AND METHODS

### 2.1 Study design

The current study was a clinical intervention, randomized, double‐blinded, placebo‐controlled study with a 6‐month follow‐up period. The study was conducted according to the principles outlined in the Declaration of Helsinki of 1975 on experimentation involving human subjects, revised in 2000. The study protocol was approved by the Human Research Ethics Committee of the Hospital of the Federal University of Rio de Janeiro (UFRJ), Brazil (approval #931.061).

### 2.2 Subject population

Participants were recruited from the Division of Graduate Periodontics of the School of Dentistry at UFRJ, between January and December of 2015. Patients were individually informed about the nature of the proposed treatment, its risks and benefits, and signed informed consent forms. To be enrolled in the study, patients had to have ≥18 years of age, ≥18 teeth, and a diagnosis of gingivitis according to Silva‐Boghossian et al. (2011); that is, >10% of sites with bleeding on probing (BOP), no probing depth (PD), and clinical attachment level (CAL) >3 mm, although PD or CAL = 4 mm in up to 5% of the sites without BOP was allowed. Exclusion criteria included history of periodontal treatment or prophylaxis 6 months previously the initial examination; smoking or history of smoking in the past 5 years; use of topical or systemic antimicrobials (including mouthwashes) in the last 6 months and of anti‐inflammatory drugs in the last 3 months prior to the initial examination; ongoing orthodontic treatment; and presence of diabetes, immune deficiencies, pregnancy, and nursing.

### 2.3 Study outcomes and sample size

The primary outcome variable was supragingival plaque, determined by the presence or absence of visible plaque (PL). Secondary outcomes included presence of gingival inflammation (GI), PD, CAL, BOP, and dental calculus index (CA). Mean prevalence and counts of microbial species were recorded as tertiary outcomes. The sample size calculation took into consideration an estimated alpha error of 5% and 80% of power to detect a 25% difference in mean reduction of percent of sites with PL between treatment groups posttherapy. This difference was calculated based on a clinical database of 124 gingivitis individuals (mean = 38.7%, standard deviation = 20.5%) regularly examined at UFRJ. Twelve subjects were required for each therapeutic group. Assuming a possible dropout rate of up to 30% throughout the study, 16 individuals were selected in each group.

### 2.4 Clinical monitoring

At the first visit, patients were evaluated regarding demographic features, medical, and dental health history by an anamnesis questionnaire. Periodontal clinical measurements were performed by a single calibrated examiner (L.C.P.E.) using a North Carolina probe (UNC‐15, Hu‐Friedy, Chicago, IL, USA). Calibration was carried out in five patients with gingivitis who were not included in the study. Pairs of examinations were conducted in each individual within 1‐week interval between them, and the kappa coefficients for PL and GI were 0.82 and 0.85, respectively. For PD and CAL, the intraclass correlation coefficients were 0.90 and 0.92. The periodontal clinical parameters evaluated included PD and CAL (mm), percent of PL, BOP, GI, and CA at six sites per tooth of all teeth, except third molars. Clinical examinations were performed at baseline (pretreatment), 1, 3, and 6 months after treatment.

### 2.5 Suitable concentration of NaOCl

In several studies, different concentrations of NaOCl have been used in oral rinse formulations (Bizzarro, Van der Velden, & Loos 2016; De Nardo et al., 2012; Galvan et al., 2014; Gonzalez et al., 2015; Lobene et al., 1972). To determine the ideal concentration of NaOCl in the mouthwash solution, we carried out a pilot test to evaluate the in vitro antimicrobial efficacy of various concentrations against subgingival plaque using a disc diffusion method. Plaque samples were obtained from patients with gingivitis, pooled, and incubated in Brain Heart Infusion broth for 48 hr at 37 °C in anaerobiosis. The plaque sample inoculum was adjusted to a 0.5 score of the McFarland scale and plated on Brain Heart Infusion agar plates. Sterile disc papers containing different concentrations of NaOCl (0.05%, 0.1%, 0.25%, 0.5%, and 1%), chlorhexidine (CHX) 0.12% (positive control), and deionized water (negative control) were placed on the plates in duplicate and incubated in anaerobiosis for 48 hr at 37 °C. Halos of inhibition measured by one examiner were 15.5 mm for CHX, 12.0 mm for 1% NaOCl, 9.5 mm for 0.5% NaOCl, 7.5 mm for 0.25% and 0.1% NaOCl, and 6.5 mm for 0.05% NaOCl. Following that, the in vivo effect of these concentrations on the reduction of salivary microbial counts was examined. Unstimulated saliva was collected from four volunteers, diluted in saline solutions and plated on blood agar. Volunteers were then asked to rinse with the solution for 30 s. Although the higher concentrations of NaOCl presented greater inhibition halos, the strong bleach smell and taste detected immediately during rinsing by the volunteers led us to test only the 0.1% solution. Saliva was again obtained 30 min after rinsing, diluted and plated on blood agar. Prerinsing and postrinsing plates were incubated for 48 hr at 37 °C in anaerobiosis. A reduction of 66.6% in colony forming units per milliliter of saliva was computed after rinsing with 0.1% NaOCl. Considering the in vitro and in vivo antimicrobial efficacy of the 0.1% NaOCl rinsing, its safety and recommendation for use as topical antimicrobial by ADA (ADA, 1984), this was the concentration selected for use as mouthwash in the present investigation.

### 2.6 Randomization and allocation concealment

Eligible subjects were allocated into a therapeutic group, T group (test) or C group (placebo), by using a block randomization method with blocks of size 4 and a sequence of random numbers. The allocation was conducted by a senior researcher (A.P.V.C.), not directly involved with the examination or treatment procedures, by using sequentially numbered opaque sealed envelopes. The codes of the groups were revealed only after completion of the statistical analyses. Mouthwashes containing 0.1% of NaOCl (T group) or distilled water (C group) were freshly prepared every week and encased in identical opaque coded bottles in the Oral Microbiology Laboratory at UFRJ. The NaOCl‐containing product was prepared by diluting a standard solution of 2.5% NaOCl (Asfer Indústria Química Ltda., SP, Brazil) in sterile distilled water. Each bottle had a total volume of 250 ml of the solution, and was weekly handled to patients. The professional (L.C.P.E.) is responsible for clinical monitoring and treatment, and all patients were blinded to treatment allocation.

### 2.7 Therapeutic protocols

After initial visit, all patients received oral hygiene instruction and a dental kit containing toothbrush, dental floss, and fluoride toothpaste. These patients returned after 1 week for treatment according to the therapeutic group they were allocated to. They received a single session of full‐mouth supra and subgingival ultrasonic debridement. Then, patients received a bottle containing 0.1% NaOCl (T group) or distilled water (C group) and were instructed to rinse with 15 ml of the product for 30 s, twice a day during 1 week. Patients returned every week with the empty bottle and received a full fresh mouthwash bottle. Mouthwashing was carried out for 4 weeks. During this time, patients were evaluated for plaque control (enhanced oral hygiene and visual inspection card) and side effects (through a specific questionnaire). Patient's adherence to therapeutic protocols was monitored by telephone messages.

### 2.8 Plaque sampling

Subgingival plaque sampling was performed at baseline, 1, 3, and 6 months after treatment. Samples were obtained from eight periodontal sites (two sites per quadrant) presenting gingivitis in each patient. After removal of supragingival plaque with sterile gauze, subgingival plaque samples were individually collected using sterile curettes (Hu‐Friedy®) and placed into microtubes containing 150 μl of TE buffer.

### 2.9 Microbiological assessment

Microbiological analyses were carried out by the checkerboard DNA–DNA hybridization technique (Socransky et al., 1994), with modifications (Heller et al., 2012). Briefly, samples were lysed and fixed in individual lanes on a nylon membrane (GE Healthcare LifeSciences, Piscataway, USA) and hybridized against whole genomic digoxigenin‐labelled (Roche Diagnóstica Brasil Ltda., São Paulo, Brazil) probes (Appendix Table 1S). DNA from *Aggregatibacter actinomycetemcomitans* serotypes a, b, and c was grouped into one probe, as was DNA from *Propionibacterium acnes* I and II. *Pantoea (*former *Enterobacter*) *agglomerans*, Enterobacter cloacae, *Enterobacter gergoviae*, *Enterobacter sakazakii*, Enterobacter aerogenes, Escherichia coli, *Klebsiella oxytoca*, and Klebsiella pneumoniae were combined in an enterics probe. Bound probes were detected with phosphatase‐conjugated antibody to digoxigenin (Roche Diagnóstica Brasil Ltda) and fluorescence (AttoPhos®, PromegaCorporation, Madison, WI) and captured by an imaging system (Storm TM 860 and ImageQuant® version 5.2, Molecular Dynamics, GE Healthcare Life Sciences). Signals were evaluated visually by comparison with the standards at 10^5^ and 10^6^ cells for the test species on the same membrane and recorded as 0 = not detected; 1 = <10^5^ cells; 2 = ~10^5^; 3 = 10^5^–10^6^ cells; 4 = ~10^6^; and 5= > 10^6^ cells.

## 3 STATISTICAL ANALYSIS

Analyses were performed using the SPSS 21.0 (IBM Brazil, SP, Brazil). Data entry was carried out by one investigator (L.C.P.E.) and error proofed by a senior investigator (A. P.V.C.). All variables were tested for normality by the Kolmogorov–Smirnov test. Demographic data were computed for each individual and compared between groups by chi‐square and Student *t* test. Periodontal measurements were averaged for each patient and within groups at each visit. Differences between groups in clinical parameters at baseline were tested by the Student *t* test, whereas differences in clinical changes over time were examined by the generalized linear model (GLM) of Repeated Measures. Microbial data were presented as mean prevalence and levels of the tested species. The frequency of each species in the eight sites was computed for each subject and then averaged within groups. Mean counts were computed for each patient and within groups. Species were also grouped into the microbial complexes (Socransky et al., 1998). Differences between groups were evaluated by chi‐square, Cochran's, Mann–Whitney, and Friedman tests. GLM was used to seek for significant differences in mean count changes of microbial complexes between groups over time. All analyses were carried out using the per‐protocol approach. For data imputation, the next observation carried backward (for 3‐month missing data) or the last observation carried forward (for 6‐month missing data) strategies were performed for missing data from patients not attending all follow‐up sessions. The level of significance was set at 5%.

## 4 RESULTS

### 4.1 Study population

**Figure 1:**
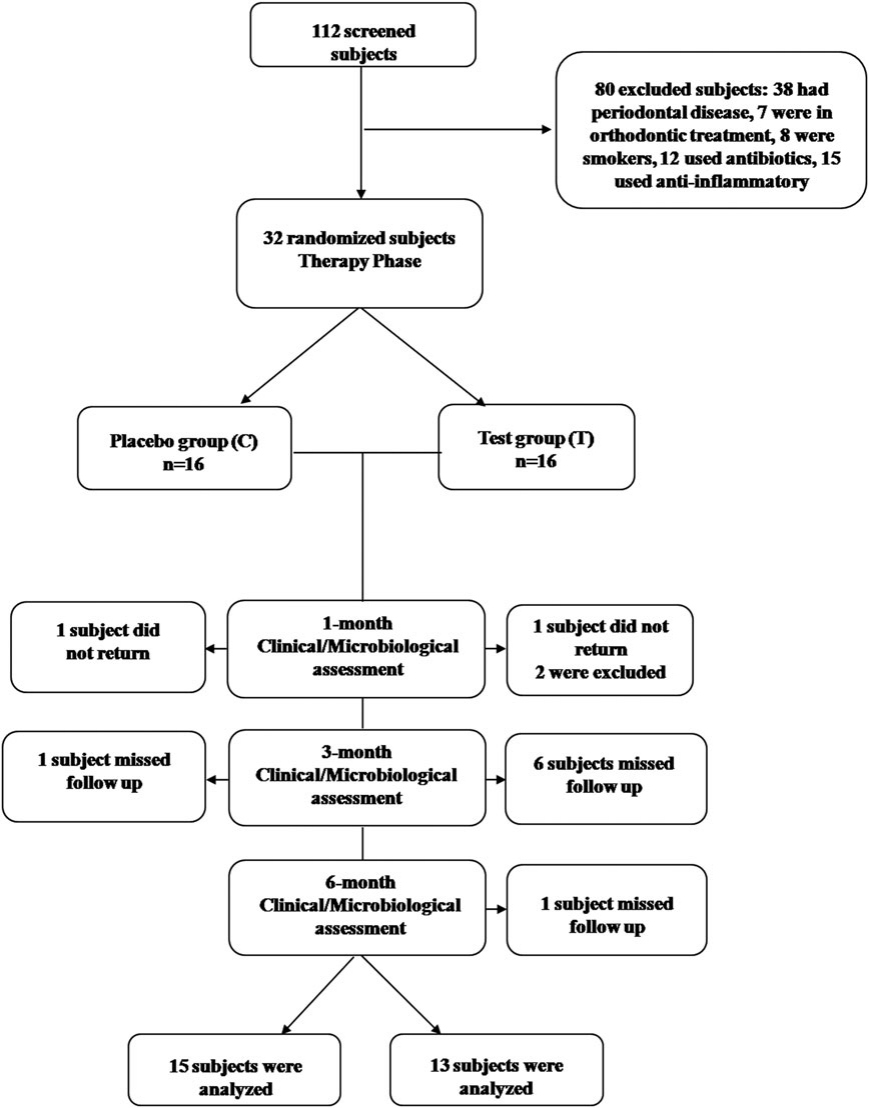
Flow chart of the study population. C = control group (full‐mouth ultrasonic debridement plus distilled water rinsing). T = test group (full‐mouth ultrasonic debridement plus 0.1% sodium hypochlorite rinsing)

The flow chart of the study population is presented in Figure 1. Out of 112 screened patients, 32 were eligible for the study based on selection criteria. All 32 patients were allocated into the two therapeutic groups and received treatment. After treatment, two patients did not return, and two were excluded due to the use of antibiotics. In addition, one patient in the C group and six patients in the T group missed the 3‐month evaluation but returned at 6 months, whereas one patient in the T group missed the 6‐month follow‐up. Thus, 28 individuals were evaluated in the current study for clinical and microbiological data.

### 4.2 Side effects

The 28 subjects who finished the study reported full adherence to the prescribed course of the placebo and NaOCl mouthwashes. Adverse effects were reported by 33.3% of the individuals in the C group and 46.2% in the T group (Appendix Table 2S). The most frequently reported side effects were bad taste (35%) and altered taste (25%). No differences between groups were observed for these side effects (chi‐square test, *p* > .05).

### 4.3 Demographic and clinical features

**Figure 2:**
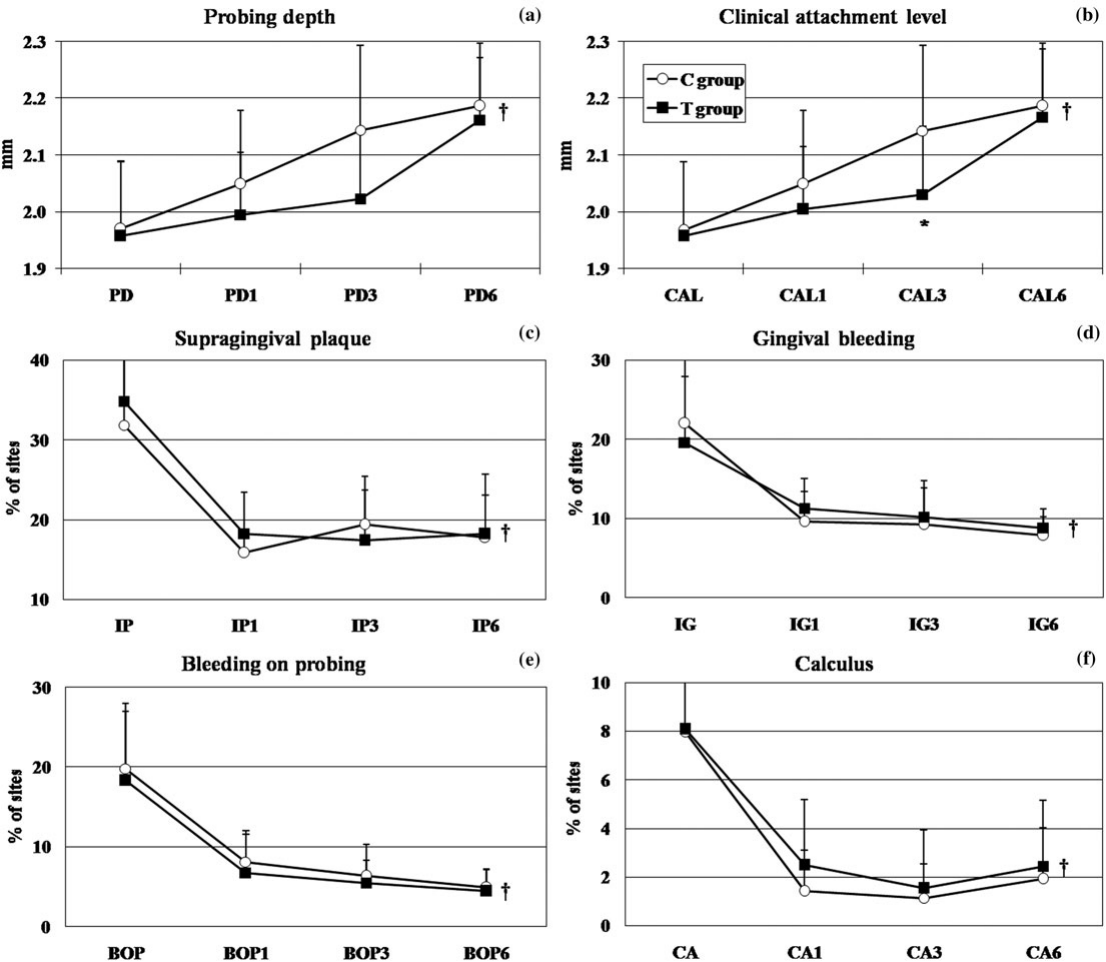
Full mouth mean (SD) of periodontal clinical parameters of individuals in both therapeutic groups at different follow up time points. (a) mean probing depth (PD; mm), (b) mean clinical attachment level (CAL; mm), mean % of sites with (c) supragingival plaque (PL), (d) gingival bleeding (GI), (e) bleeding on probing (BOP), and (f) calculus (CA). C group = full‐mouth ultrasonic debridement + distilled water rinsing; T group = full‐mouth ultrasonic debridement + 0.1% sodium hypochlorite rinsing. * refers to significant difference between groups at 3 months, *p* < .05 (*t* test). † refers to significant differences within groups over time (*p* < .01, generalized linear model [GLM]). Both groups showed significant changes over time for this parameter, but no differences between groups were observed (GLM test)

Baseline demographic and clinical data of the study population are presented in Table 1. Similar distributions for gender, race or color and socioeconomic levels, and mean age were observed for both therapeutic groups at baseline. Likewise, both groups presented similar periodontal clinical conditions of gingival inflammation and oral hygiene. Regarding therapeutic response, Figure 2 shows the periodontal clinical parameters in both groups posttherapy. For PD (Figure 2A) and CAL (Figure 2B), there was a significant but modest increase (0.22 mm for C and 0.20 mm for T group) in both groups over time (Friedman test, *p* < .01). This increase was slower in the T group, and at 3 months, the mean PD and CAL was significantly higher in the C group compared to that in the T group (*t* test, *p* < .05). Both groups showed significant reductions in PL, GI, CA, and BOP (Figures 2C–F) over time (Friedman test, *p* < .01), but these differences were not significant between groups (GLM test, *p* < .05).

**Table 1:** Demographic and clinical periodontal data of the sample population at baseline.

Parameters	C group (*n* = 15)	T group (*n* = 13)
Mean (SD) age (years)	25.1 (6.8)	24.7 (5.3)
Gender		
% males	26.7	30.8
% females	73.7	69.2
Race		
% white	66.7	76.9
% African American	6.7	7.7
% others	26.6	15.4
% monthly family income (US$)		
≤500	20.0	15.4
>500	80.0	84.6
% education		
High school	7.0	8.0
Higher education	93.0	92.0
Mean (SD) periodontal parameters		
Missing teeth	0.6 (1.4)	1.0 (3.0)
PD (mm)	1.9 (0.1)	1.9 (0.1)
CAL (mm)	1.9 (0.1)	1.9 (0.1)
% sites with BOP	19.8 (8.2)	18.3 (8.7)
% sites with PL	31.8 (8.9)	34.8 (17.2)
% sites with GI	22.0 (8.3)	19.6 (6.9)
% sites with CA	8.0 (5.6)	8.1 (8.2)

*Note*. C group = control group (full‐mouth ultrasonic debridement + distilled water rinsing); T group = test group (full‐mouth ultrasonic debridement + 0.1% NaOCl rinsing). PD = probing depth; CAL = clinical attachment level; BOP = bleeding on probing; PL = supragingival plaque; GI = gingival bleeding; CA = calculus index; SD = standard deviation.

### 4.4 Microbiological data

**Figure 3:**
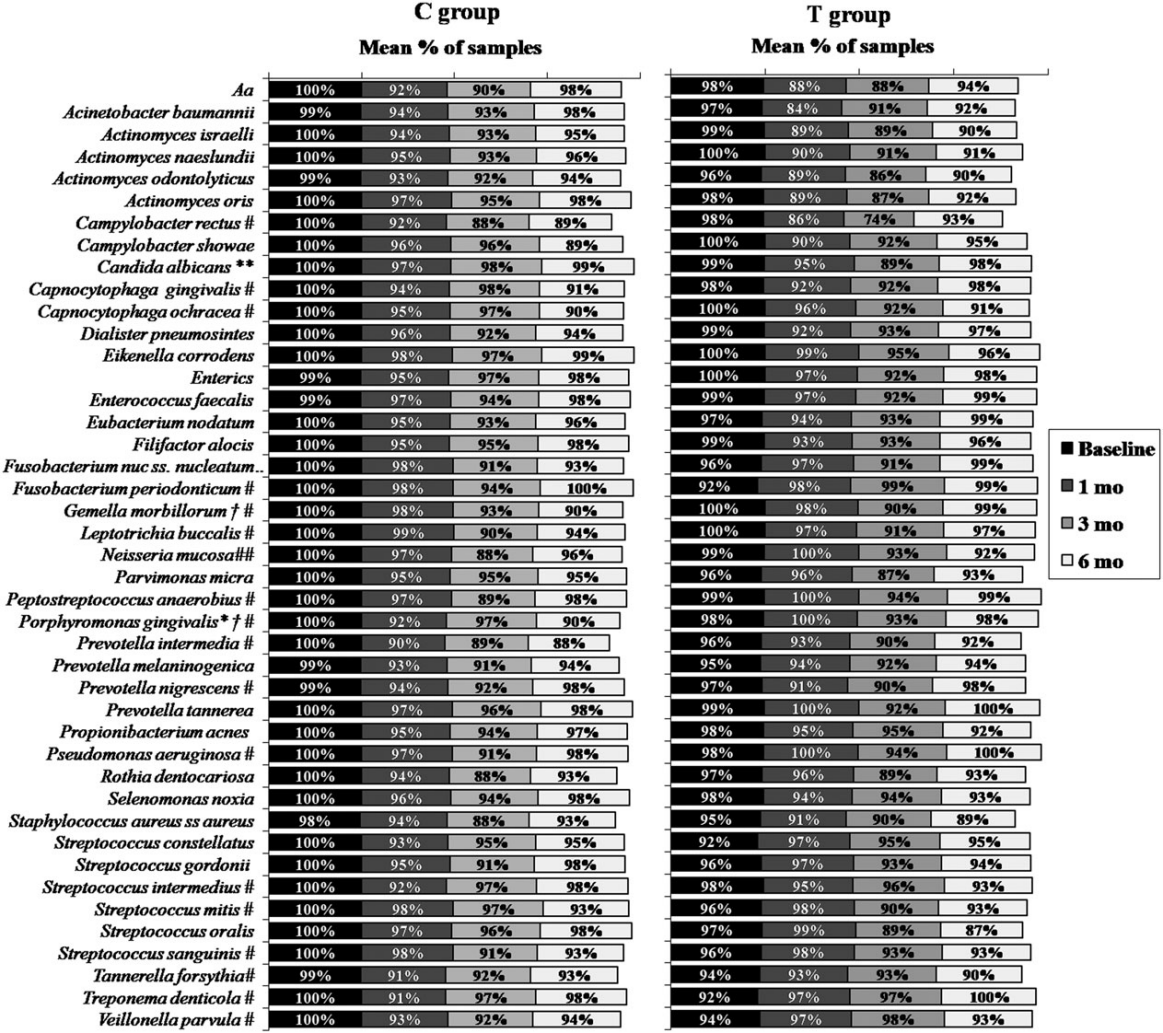
Mean frequency (%) of microbial species in subgingival plaque samples from both therapeutic groups at baseline and different posttherapy time points. C group = full‐mouth ultrasonic debridement + distilled water rinsing; T group = full‐mouth ultrasonic debridement + 0.1% sodium hypochlorite rinsing. *Aa* = *Aggregatibacteractinomycetemcomitans*. * refers to significant differences between groups at 1 month, ** at 3 months, and † at 6 months (*p* < .05, Mann–Whitney test). # refers to significant changes over time within the C group, and ## within the T group (*p* < .05, Friedman test)

Changes in prevalence of microbial species over time are presented in Figure 3. Significant reductions were observed in the C group for several species, however, only *Neisseria mucosa* decreased significantly in the T group at 6 months (*p* < .05, Friedman test). Differences between groups were observed for Candida albicans, *Fusobacterium nucleatum ss. nucleatum*, *Gemella morbillorum*, and *Porphyromonas gingivalis* after therapy (*p* < .05, Mann–Whitney test). Considering that eight periodontal sites were sampled per patient, very little changes in the colonization of these sites by the tested species were observed after treatments.

**Figure 4:**
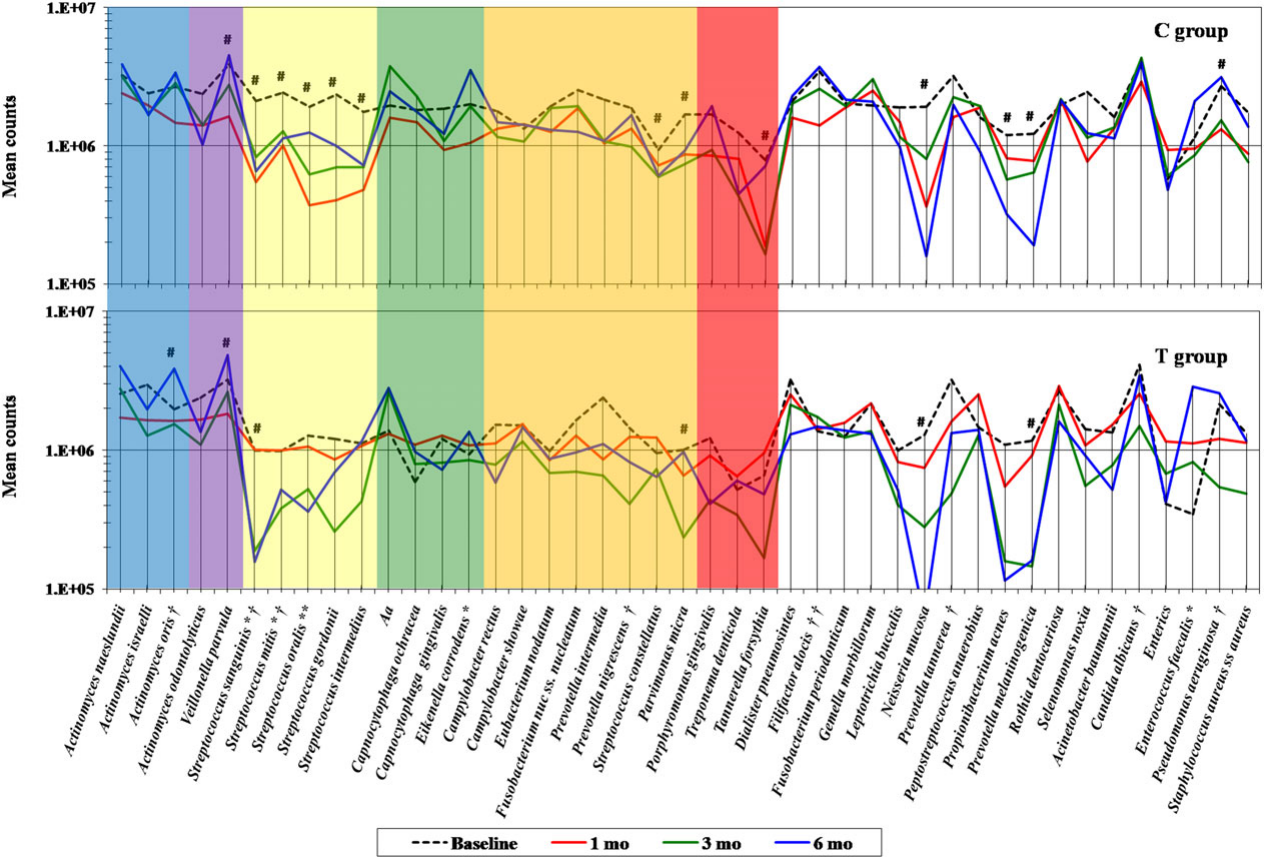
Mean counts of microbial species in subgingival plaque samples from both therapeutic groups at baseline and different posttherapy time points. C group = full‐mouth ultrasonic debridement + distilled water rinsing; T group = full‐mouth ultrasonic debridement + 0.1% sodium hypochlorite rinsing. *Aa* = *Aggregatibacteractinomycetemcomitans*. The colored shades represent the microbial complexes (Socransky et al., 1998; Socransky & Haffajee, 2002). * refers to significant differences between groups at baseline, ** at 1 month, † at 3 months, and †† at 6 months (*p* < .05, Mann–Whitney test). # refers to significant changes in microbial counts over time within each group (*p* < .05, Friedman test)

Reductions in mean counts were more pronounced and were detected in more than 67% of the species evaluated in both groups at 6 months (Figure 4). Changes were significant for several species in the C group, whereas only six species (*Actinomyces oris*, *Veillonella parvula*, *Streptococcus sanguinis*, *Parvimonas micra*, *Neisseria mucosa*, and *Prevotella melaninogenica*) showed significant changes over time in the T group. V. parvula increased significantly in mean counts in both groups; however, oral streptococci showed a mean reduction posttherapy, particularly in the C group. Other health‐related species (*A*. *oris*) increased significantly in the T group (*p* < .05, Friedman test). Comparisons between groups at the posttherapy follow‐ups showed significant differences mostly at 1 and 3 months. At 6 months, only *Filifactor alocis* was detected in significantly lower levels in the T than in the C group (*p* < .05, Mann–Whitney test).

**Figure 5:**
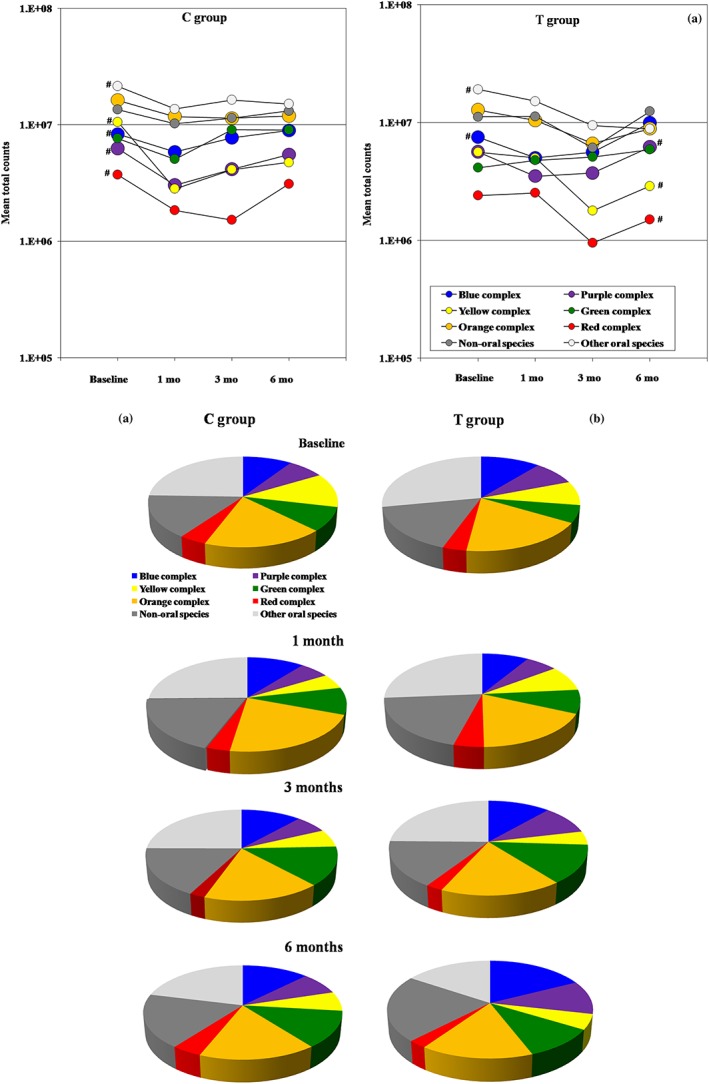
(a) Mean total counts of microbial members of the oral complexes (Socransky et al., 1998; Socransky & Haffajee, 2002) in subgingival plaque of individuals from both C group (full‐mouth ultrasonic debridement + distilled water rinsing) and T group (full‐mouth ultrasonic debridement + 0.1% sodium hypochlorite rinsing) over time. (b) Proportions of each microbial complex of the total mean counts of species from all complexes. # refers to significant changes in microbial counts of the complexes over time within groups (*p* < .05, generalized linear model test)

Species were clustered into microbial complexes (Socransky et al., 1998), as well as other two cluster of nonoral opportunist species and other oral microorganisms. The mean counts of each member of a complex were added within that complex. The proportion of each complex of the total microbial counts including all species was also computed. Significant changes in the mean total counts of the complexes over time (Figure 5A) were observed for the purple, blue, yellow, and red complexes and other oral species (*p* < .05, GLM). These changes were similar between groups (*p* > .05, GLM). Complexes reduced in counts mainly right after the mouthwash use (1 month) and at 3 months but returned to baseline levels at 6 months in both groups, with the exception of the red and yellow complexes and other oral species, which were kept at low levels over time (*p* < .05, GLM). In terms of proportions (Figure 5B), there was an increase in the blue, purple, and green complexes and in nonoral species. In contrast, orange and yellow complexes and other oral species decreased in proportions of the total counts over time. These changes were more pronounced in the T group, but no differences between groups were observed. Of interest, the red complex diminished in proportions in the T group over time, and this complex returned to baseline proportions at 6 months posttherapy in the C group.

## 5 DISCUSSION

The current randomized clinical trial evaluated the efficacy of NaOCl mouthwash as an adjunct to FMUD on the reduction of supragingival plaque, gingival inflammation, and levels of subgingival microbial species in gingivitis patients for a period of 6 months. Our data showed no additional benefits of NaOCl mouthwash over FMUD on the clinical or microbiological parameters evaluated. One initial concern about the use of this antiseptic was the ideal concentration, because various empiric concentrations have been used. We have chosen the 0.1% concentration based on in vitro and in vivo pilot experiments and on the recommendation of the ADA (ADA, 1984). Other investigators have proposed higher concentrations (0.25%), however, as a twice a week mouthwash without any professional mechanical intervention (Galvan et al., 2014; Gonzalez et al., 2015). These authors showed a greater efficacy of NaOCl rinsing compared to placebo, but there was also a significant increase in dropout rate (40%). Bizzarro et al. (2016) showed no side effects and a small dropout rate (10%), even when using a high concentration of NaOCl (0.5%) over 1 year; however, the antimicrobial was used as an intrapocket irrigation solution. In our study, the overall dropout rate was relatively low (12.5%). Moreover, the adverse effects were considered tolerable, and as expected, bad taste was the most common side effect here reported (De Nardo et al., 2012; Galvan et al., 2014; Gonzalez et al., 2015). Thus, it is possible that at this low concentration (0.1%), NaOCl does not present the best antimicrobial efficacy against the periodontal microbiota, which would justify our clinical and microbiological findings. Nevertheless, we feel that higher concentrations of NaOCl for long‐term daily use as a mouthwash would result in significant more side effects and consequently less adherence to treatment.

Our clinical data showed that gingivitis patients from both therapeutic groups presented a significant reduction in supragingival plaque, periodontal inflammation, and calculus over time, and the use of 0.1% NaOCl mouthwash for 4 weeks did not provide an additional benefit to FMUD. Conversely, other authors have shown a greater and significant reduction in supragingival plaque accumulation and gingivitis in the NaOCl group compared to the distilled water placebo group (De Nardo et al., 2012; Galvan et al., 2014; Gonzalez et al., 2015). These contradictory data may be explained by differences in NaOCl concentration, study design, periodontal clinical condition of the sample population, as well as the administration, frequency, and duration of the antimicrobial (De Nardo et al., 2012; Galvan et al., 2014; Gonzalez et al., 2015; Lobene et al., 1972). Two of these studies used an experimental gingivitis model, and the NaOCl was not used as an adjunct to mechanical plaque control but as a substitute of oral hygiene methods. In this scenario of absence of oral hygiene, rinsing with NaOCl seems to prevent new plaque formation De Nardo et al., 2012; Lobene et al., 1972). Others have reported an efficient anti‐inflammatory effect of a higher concentration of NaOCl rinsing (0.25%) in individuals with periodontitis. A significant reduction in BOP, even in moderate to deep periodontal pockets, was observed in the NaOCl group compared to placebo in the absence of supra or subgingival scaling and root planing (Galvan et al., 2014; Gonzalez et al., 2015). The authors speculate that the anti‐inflammatory effect of oral rinsing on deep pockets may have resulted from the antiplaque effect of NaOCl on supragingival plaque accumulation, which in turn would lead to beneficial changes in the composition of the subgingival microbiota (Carvalho et al., 2004; Gonzalez et al., 2015; Haffajee et al., 2006, Ximenez‐Fyvie et al., 2000). In addition, NaOCl itself has been shown to exert anti‐inflammatory properties by interfering on activation of proinflammatory genes (Leung et al.,^2013^; Mainnemare et al., 2004). The only clinical study that did not demonstrate a greater clinical and antimicrobial efficacy of 0.5% NaOCl over basic periodontal therapy used this antiseptic as a one‐session intrapocket irrigation solution (Bizzarro, Van der Velden, & Loos, 2016). Moreover, in their study design, all patients rinsed twice a day with 0.12% CHX for 28 days previously to the beginning of mechanical periodontal treatment. These data should be considered carefully because topical antimicrobials, either as a mouthwash or as a periodontal irrigation solution, should be administered as an adjunct to mechanical therapy, particularly in periodontitis patients. In addition to low concentration, lack of efficacy of NaOCl mouthwash observed in our study may be attributed to the short period of use (4 weeks). However, there are no consensus regarding the ideal duration of rinsing for most mouthwashes, and the effects of a long‐term use of NaOCl mouthwash have not been evaluated in any of these studies.

Although there was a significant improvement in terms of dental plaque and periodontal inflammation, a significant but clinically minimal (0.2 mm) increase in mean PD and CAL was observed in both groups over time. According to previous studies, shallow sites do lose attachment (approximately 0.4 mm) after mechanical instrumentation, whereas greater attachment gains are observed in deep pockets (Cobb, 1996; Faveri et al., 2006; Hung & Douglass, 2002; Van der Weijden & Timmerman, 2002). Given that our gingivitis patients presented mostly 1‐ to 3‐mm PD, these findings could be expected.

In our current “highly germ‐free era,” it is important to understand the short‐ and long‐term impacts of widely used topical antiseptics on the diversity of our microbiome (Cho & Blaser, 2012; Reid et al., 2011). After therapy, most species in the subgingival plaque presented a modest decrease in prevalence in both groups. As reported by other investigators (Colombo et al., 2005; Cugini et al., 2000; Haffajee et al., 1997), nonsurgical mechanical periodontal therapy has a less pronounced impact on the frequency than on the counts of microbial species in the periodontal microbiota. This fact reflects the characteristic resilience of our oral microbiota, which tend to rebound and recolonize the same oral habitats after a disturbance such as mechanical debridement and/or topical antimicrobial (Filoche, Wong, & Sissons, 2010). However, posttherapy recolonization normally occurs at levels lower than the pretherapy levels. In fact, over 65% of the tested species here evaluated were detected in lower counts at 6 months in both groups, including periodontal pathogens but also some periodontal health‐related species such as oral streptococci and N. mucosa. The opportunist pathogens *Enterococcus faecalis* and *Pseudomonas aeruginosa* were present in higher counts at 6 months in both groups, whereas Gram‐negative enterics increased after therapy and then returned to pretherapy counts in both groups. The increase in levels of these microorganisms in the subgingival plaque may be a result of the reduction of several other oral bacteria, which may have opened a new niche for these species. At the final evaluation (6 months), only *F*. *alocis* was detected in higher counts in the C compared to the T group. This species has been considered a major periodontal and endodontic pathogen associated with persistent infection (Colombo et al., 2009; Siqueira Jr & Rôças, 2009), and its reduction should be considered as a target for treatment with antimicrobials.

Clustering of subgingival plaque species into microbial complexes also revealed similar changes in both groups over time. Except for streptococci, health‐related complexes increased at 6 months, particularly in the T group. Furthermore, the pathogenic red complex had a more pronounced reduction in the T group and it showed a tendency to return to baseline levels in the placebo group. In general, changes over time on the microbial profile of the subgingival microbiota were quite even between groups, corroborating the similarity in the clinical response to both treatments.

Despite the fact that NaOCl is an efficient, safe, and low cost antiseptic, its short‐term use as a mouthwash, at the recommended concentration of 0.1%, does not provide additional clinical and/or microbiological benefits to FMUD in individuals with gingivitis. Further clinical investigations testing higher concentrations and/or longer periods of administration are encouraged to determine whether this antimicrobial may indeed be recommended as a long‐term daily mouthwash to chemical plaque control.

## CONFLICT OF INTEREST AND SOURCE OF FUNDING

The authors declare that they have no conflict of interests. This work was supported in part by National Council for Scientific and Technological Development (CNPq); Coordination of Improvement of Higher Education Personnel (CAPES), Brasilia, Brazil; and Foundation for Research Financial Support in the State of Rio de Janeiro (FAPERJ), Rio de Janeiro, Brazil.

## Supporting information


**Appendix Table 1S.** Microbial strains used for the whole genomic DNA probes.
**Appendix Table 2S.** Adverse effects reported in by individuals from both therapeutic groups.
