# Supplementary material for: Lack of adjunctive effect of 0.1% sodium hypochlorite mouthwash combined to full‐mouth ultrasonic debridement on supragingival plaque, gingival inflammation, and subgingival microbiota: A randomized placebo‐controlled 6‐month trial
Source: Clin Exp Dent Res. 2017 Mar 31;3(2):51–61. doi: 10.1002/cre2.60 (PMC5719817; doi:10.1002/cre2.60)
Supplement: Supplementary file 1 — Appendix Table 1S. Microbial strains used for the whole genomic DNA probes. Appendix Table 2S. Adverse effects reported in by individuals from both therapeutic groups. [file CRE2-3-51-s001.doc]

**Appendix**

**Appendix Table 1S.**Microbial strains used for the whole genomic DNA probes.

| **Taxa** | **Strain^a^** | **Taxa** | **Strain^a^** |
| --- | --- | --- | --- |
| *Aggregatibacter actinomycetemcomitans a* | 43718 | *Gemella morbillorum* | 27824 |
| *Aggregatibacter actinomycetemcomitans b* | 29523 | *Leptotrichia buccalis* | 14201 |
| *Aggregactibacter actinomycetemcomitans c* | 625^b^ | *Klebsiella pneumoniae* | 10031 |
| *Acinetobacter baumannii* | 19606 | *Klebsiella oxytoca* | 12833 |
| *Actinomyces israelli* | 12102 | *Neisseria mucosa* | 19696 |
| *Actinomyces odontolyticus* | 17929 | *Parvimonas micra* | 33270 |
| *Actinomyces naeslundii I* | 12104 | *Peptostreptococcus anaerobius* | 27337 |
| *Actinomyces oris(A.viscosus)* | 43146 | *Prevotella melaninogenica* | 25845 |
| *Campylobacter rectus* | 33238 | *Porphyromonas gingivalis* | 33277 |
| *Capnocytophaga gingivalis* | 33624 | *Prevotella intermedia* | 25611 |
| *Capnocytophaga ochracea* | 33596 | *Prevotella nigrescens* | 33563 |
| *Candida albicans* | 10231 | *Prevotellatannerae* | 51259 |
| *Campylobacter showae* | 51146 | *Pseudomonas aeruginosa* | 10145 |
| *Dialisterpneumosintes* | GBA27^b^ | *Propionibacterium acnes I* | 11827 |
| *Eubacterium nodatum* | 33099 | *Propionibacterium acnes II* | 11828 |
| *Eikenella corrodens* | 23834 | *Rothiadentocariosa* | 17931 |
| *Enterococcus faecalis* | 10100 | *Selenomonas noxia* | 43541 |
| *Enterobacter agglomerans* | 27155 | *Streptococcus constellatus* | 27823 |
| *Enterobacter cloacae* | 10699 | *Streptococcus mitis* | 49456 |
| *Enterobacter sakazakii* | 12868 | *Streptococcus oralis* | 35037 |
| *Enterobacter aerogenes* | 13048 | *Streptococcus sanguinis* | 10556 |
| *Enterobacter gergoviae* | 33028 | *Streptococcus gordonii* | 10558 |
| *Escherichia coli* | 10799 | *Streptococcus intermedius* | 27335 |
| *Filifactoralocis* | 35896 | *Staphylococcus aureus ss aureus* | 33591 |
| *Fusobacterium nucleatum ss. nucleatum* | 25586 | *Tannerella forsythia* | 43037 |
| *Fusobacterium periodonticum* | 33693 | *Treponema denticola* | B1^b^ |
|  |  | *Veillonella parvula* | 10790 |

^a^ ATCC (American Type Culture Collection, Rockville, MD); ^b^ The Forsyth Institute, Cambridge, MA

**Appendix Table 2S.** Adverse effects reported in by individuals from both therapeutic groups.

| **Side Effects** | **C group**  **(n=15)** | **T group**  **(n=13)** |
| --- | --- | --- |
| No adverse effects reported | 10 | 7 |
| Bad taste | 4 | 6 |
| Altered taste | 3 | 4 |
| Burning sensation | 1 | 3 |
| Ulcers | 3 | 1 |
| Nausea | 1 | 2 |
| Tooth whitening | 1 | 1 |

C group (full-mouth ultrasonic debridement + distilled water rinsing) and T group (full-mouth ultrasonic debridement + 0.1% NaOCl rinsing)
